# Information gain on variable neuronal firing rate

**DOI:** 10.1186/1471-2202-14-S1-P221

**Published:** 2013-07-08

**Authors:** Shinsuke Koyama

**Affiliations:** 1 The Institute of Statistical Mathematics, Tokyo 190-8562, Japan

## 

The question of how much information can be theoretically gained from variable neuronal firing rate with respect to constant mean firing rate is investigated. For this purpose, we employ the Kullback-Leibler divergence as a measure of information gain.

We first give a statistical interpretation of this information in terms of detectability of rate variation: the lower bound of detectable rate variation, below which the temporal variation of firing rate is undetectable with a Bayesian decoder, is entirely determined by this information [1]. We derive a formula for the lower bound, which tells how much information is necessary for the rate variation to be detected from spike trains. For instance, if a spike, on average, is expected to be observed in the characteristic timescale of the rate variation, it is necessary for the spike train to carry more than 0.36 bits per spike information so that the underlying rate variation is detectable.

We show that the information depends not only of the variation of firing rates (i.e., signals), but also significantly on the dispersion properties of neuronal firing described by the shape of interspike interval (ISI) distribution (i.e., noise properties). It is shown that under certain condition, the gamma distribution attains the theoretical lower bound of the information among all ISI distributions when the coefficient of variation of ISIs is given [2]. We also give a useful formula for the (approximate) information, which provides a theoretical prediction for the range of the information gain.

With the basis of the theoretical investigations, we propose a practical procedure for estimating the information from spike trains, and apply this method to biological spike data recorded from a cortical area. The estimated information ranges up to 0.8 bits/spike, which roughly matches the theoretical prediction.
